# Overexpression of S100A4 in human cancer cell lines resistant to methotrexate

**DOI:** 10.1186/1471-2407-10-250

**Published:** 2010-06-01

**Authors:** Nuria Mencía, Elisabet Selga, Isabel Rico, M Cristina de Almagro, Xenia Villalobos, Sara Ramirez, Jaume Adan, Jose L Hernández, Véronique Noé, Carlos J Ciudad

**Affiliations:** 1 Department of Biochemistry and Molecular Biology, School of Pharmacy, University of Barcelona, Diagonal Avenue 643, Barcelona, Spain; 2 Leitat Technological Center (Biomed Division), Helix building PCB, Barcelona, Spain

## Abstract

**Background:**

Methotrexate is a chemotherapeutic drug that is used in therapy of a wide variety of cancers. The efficiency of treatment with this drug is compromised by the appearance of resistance. Combination treatments of MTX with other drugs that could modulate the expression of genes involved in MTX resistance would be an adequate strategy to prevent the development of this resistance.

**Methods:**

The differential expression pattern between sensitive and MTX-resistant cells was determined by whole human genome microarrays and analyzed with the GeneSpring GX software package. A global comparison of all the studied cell lines was performed in order to find out differentially expressed genes in the majority of the MTX-resistant cells. S100A4 mRNA and protein levels were determined by RT-Real-Time PCR and Western blot, respectively. Functional validations of S100A4 were performed either by transfection of an expression vector for S100A4 or a siRNA against S100A4. Transfection of an expression vector encoding for β-catenin was used to inquire for the possible transcriptional regulation of S100A4 through the Wnt pathway.

**Results:**

S100A4 is overexpressed in five out of the seven MTX-resistant cell lines studied. Ectopic overexpression of this gene in HT29 sensitive cells augmented both the intracellular and extracellular S100A4 protein levels and caused desensitization toward MTX. siRNA against S100A4 decreased the levels of this protein and caused a chemosensitization in combined treatments with MTX. β-catenin overexpression experiments support a possible involvement of the Wnt signaling pathway in S100A4 transcriptional regulation in HT29 cells.

**Conclusions:**

S100A4 is overexpressed in many MTX-resistant cells. S100A4 overexpression decreases the sensitivity of HT29 colon cancer human cells to MTX, whereas its knockdown causes chemosensitization toward MTX. Both approaches highlight a role for S100A4 in MTX resistance.

## Background

Methotrexate (MTX) is a classical drug that is used for the treatment of a wide variety of cancers, both alone and in combination with other chemotherapeutic agents [1,2]. Drug resistance is usually observed upon treatment with MTX, thus compromising its effectiveness. Combination treatments of MTX with other drugs that could modulate the expression of genes involved in MTX resistance would be an adequate strategy to prevent the development of resistance. With this premise, we searched for genes differentially expressed in common among seven MTX-resistant cell lines representative of five different types of cancer. In that work we identified and validated DHFR as the only gene overexpressed in common among all the studied cell lines [3]. We also identified and validated other genes that, aside of DHFR, played an important role in MTX resistance (AKR1C1, DKK1) [4] or that indirectly contributed to the resistance phenotype (MSH3, SSBP2, ZFYVE16) [5].

In the present report, we identified genes differentially expressed in at least 4 out of the seven cell lines. Among the genes that fulfilled this requisite, we found some genes that we had previously studied as modulators of MTX resistance, and S100A4, a gene overexpressed in five out of the seven cell lines studied.

S100A4 is a member of the S100 calcium binding protein family, which is composed of more than 20 members. Their name was given because they are soluble in 100% saturated ammonium sulfate [6]. As many of the S100 family members, S100A4 is a symmetric homodimer characterized by the presence of two calcium binding sites of the EF-hand type (helix-loop-helix) [7] that enable S100 proteins to respond to calcium stimulus induced by cell signaling.

S100A4 has been described to be involved in a wide variety of intra- and extracellular processes, such as protein phosphorylation, dynamics of cytoskeleton components or Ca^2+ ^homeostasis, which are regulated through interaction of S100A4 with its target proteins [8,9]. Overexpression of S100A4 has been associated with tumor malignancy [10] as well as to metastasis [11], angiogenesis [12] and chemoresitance [13].

In this work, we searched for genes differentially expressed in common among cell lines resistant to MTX. We identified S100A4 as a gene overexpressed in five out of the seven MTX-resistant cell lines studied, which had been previously associated with chemotherapy resistance. Functional validations using either an expression vector encoding for S100A4 or siRNA against its RNA show a role for S100A4 in MTX resistance.

## Methods

### Cell Lines

Cell lines representative of five types of human cancer were used: HT29 and Caco-2 of colon cancer, MCF-7 and MDA-MB-468 of breast cancer, MIA PaCa-2 of pancreatic cancer, K562 of erythroblastic leukemia, and Saos-2 of osteosarcoma [3]. Resistant cells were obtained in the laboratory upon incubation with stepwise concentrations of MTX (Lederle) as previously described [4]. HT29, Caco-2 and K562 resistant cells were able to grow in 10^-5^M MTX, which corresponds to a 1000-fold increase in resistance with respect to their respective sensitive cells; MIA PaCa-2, Saos-2, MCF-7 and MDA-MB-468 were resistant to 10^-6^M MTX, which represents an increase in resistance of 100 times with respect to their sensitive counterparts.

### Cell Culture

Human cell lines were routinely grown in Ham's F12 medium supplemented with 7% fetal bovine serum (FBS, both from Gibco) at 37°C in a 5% CO_2 _humidified atmosphere. Resistant cells were routinely grown in selective DHFR medium (-GHT medium, GIBCO) lacking glycine, hypoxanthine and thymidine, the final products of DHFR activity. This medium was supplemented with 7% dialyzed fetal bovine serum (GIBCO).

### Global microarray data analyses of cell lines resistant to MTX

A global comparison of all cell lines was performed using GeneSpring GX v 7.3.1 (Agilent Technologies), using the latest gene annotations available (March 2009), in order to find differentially expressed genes in the majority of the resistant cell lines. The triplicate samples for each condition, sensitive and resistant, in each of the seven cell lines (42 samples in total) were imported into one single experiment. Normalization was applied in two steps: i) "per Chip normalization" by which each measurement was divided by the 50th percentile of all measurements in its array; and ii) "per Gene normalization" by which the samples of each cell line were normalized against the median of the respective sensitive cells (control). The expression of each gene was calculated as the ratio of the value obtained for each condition relative to the control condition after normalization of the data. Then, data were filtered using the control strength, a control value calculated using the Cross-Gene Error Model on replicates [14] and based on average base/proportional value. Measurements with higher control strength are relatively more precise than measurements with lower control strength. Genes that did not reach this value were discarded. Additional filtering was performed to determine the differentially expressed genes. We selected the genes that displayed a p-value corrected by false discovery rate (Benjamini and Hochberg FDR) of less than 0.05. A list was generated with the genes that passed those filters in at least four out of the seven resistant cell lines.

### RT-Real-Time PCR

mRNA levels were determined by RT-Real-time PCR. Total RNA was extracted from cells using Ultraspec™ RNA reagent (Biotecx) following the recommendations of the manufacturer. Complementary DNA was synthesized in a total volume of 20 μl by mixing 500 ng of total RNA, 125 ng of random hexamers (Roche), in the presence of 75 mM KCl, 3 mM MgCl_2_, 10 mM dithiothreitol, 20 units of RNasin (Promega), 0.5 mM dNTPs (AppliChem), 200 units of M-MLV reverse transcriptase (Invitrogen) and 50 mM Tris-HCl buffer, pH 8.3. The reaction mixture was incubated at 37°C for 60 min. The cDNA product was used for subsequent Real-time PCR amplification using an ABI Prism 7000 Sequence Detection System (Applied Biosystems) with 25 ng of the cDNA mixture and the assays-on-demand from Applied Biosystems Hs00243202_m1 for S100A4 and Hs00356991_m1 for APRT.

### Gene copy number determination

Genomic DNA from either sensitive or resistant cells was obtained with the Wizard™ Genomic DNA Purification Kit (Promega) following the manufacturer's recommendations. We used 25 ng of DNA and the following primers for Real-Time PCR amplification: For S100A4: 5'-CTTCTGG-GCTGCTTAT-3' and 5'-ACTGGGCTTCTGT-TTTCTATC-3'; and for APRT: 5'-CGGGAAC-CCTCGTCTTTCGCC-3' and 5'-GCCTCGGG-GGCTCAATCTCAC-3'.

### Preparation of total extracts for Western blotting

Total extracts from cells were used to assay S100A4 protein levels. Cells were scrapped in 700 μl of ice-cold PBS and centrifuged for 10 min at 2,000 rpm on a microfuge. The supernatant was discarded and cells were resupended in 50 μl of RIPA buffer (50 mM Tris-HCl, 150 mM NaCl, 5 mM EDTA, 1% Igepal CA-630 (Sigma), 0.5 mM PMSF, Protease inhibitor cocktail from Sigma, 1 mM NaF; pH 7.4). Cells were incubated in ice for 30 min with vortexing every 10 minutes and then centrifuged at 15,000 × g at 4°C for 10 min. Five μl of the extract were used to determine protein concentration by the Bradford assay (Bio-Rad). The extracts were frozen in liquid N_2 _and stored at -80°C. Different amounts of total extracts were resolved on SDS 15%-polyacrylamide gels. After protein transference using a semidry electroblotter, PVDF membranes (Immobilon P, Millipore) were incubated with an antibody against S100A4 (DAKO), and detection was accomplished by secondary horseradish peroxidase-conjugated antibody and enhanced chemiluminescence, as recommended by the manufacturer (Amersham). To normalize the results, blots were re-probed with antibodies against either Actin or GAPDH (Sigma).

### Transfection of an expression vector encoding for S100A4

Cells were seeded into 6-well plates in 1 ml of HAM's F12 selective medium. Eighteen hours later, cells were transfected with the expression plasmid for S100A4 (pCMV6-XL5-S100A4, OriGene Technologies; abbreviated in the manuscript as pCMV-S100A4), using either Lipofectamine™2000 (Invitrogen) or Fugene (Roche) following the manufacturer's specifications. The overexpression of S100A4 was monitored by determining its mRNA levels after 48 h and by determining its protein levels after 72 h upon transfection, respectively. When assaying the sensitivity to MTX caused by overexpression of S100A4, MTX was added 48 h upon transfection and cell viability determined using the MTT assay 6 days after MTX addition. An empty vector was transfected in parallel with pCMV-S100A4 and was used as negative control.

### ELISA assay

Cells were transfected with pCMV-S100A4 as described above and cell culture media was collected 72 h after transfection. Sandwich ELISA was performed in MaxiSorb plates (NUNC), coated with a mouse anti-S100A4 antibody O/N at 4°C. Blocking was performed with 1% BSA for 1 h at 37°C. Incubation with the samples was performed for 2.5 h at 37°C, followed by incubation with a rabbit anti-S100A4 antibody. Finally, incubation with a secondary goat anti-rabbit horseradish peroxidase-conjugated antibody was performed for 1 h at 37°C. Reading of absorbance upon TMB addition was performed in a Multiskan Ascent (Thermo) plate-reading spectrophotometer at 630 nm. Background signal was determined in parallel and was used to correct the absorbance values. All analyses were performed at least in duplicate for each sample in each experiment.

### Transfection of siRNAs against S100A4 RNA

HT29 cells were plated in 1 ml of -GHT medium. Transfection was performed eighteen hours later with a siRNA designed against S100A4 RNA (siS100A4) or with a siS100A4-4 MIS bearing 4 mismatches with respect to siS100A4 (underlined in the sequence below).

siS100A4: 5'-CAGGGACAACGAGGTGGAC-3'

siS100A4-4 MIS: 5'-CACCCTCAACGAGGTGGAC-3'

Cells were lipofected with the siRNAs using Lipofectamine™2000 (Invitrogen) in accordance to the manufacturer's instructions. MTX (10^-7^M) was added 48 hours after siRNA treatment and MTT assays were performed as described previously [15] after 5 days from the treatment with the siRNA. S100A4 knockdown was monitored by determining its mRNA levels after 48 h and its protein levels after 72 h upon siRNA transfection, respectively.

The siRNAs were designed using the software iRNAi v2.1. Among the possible alternatives, sequences rich in A/T on the 3' of the target were chosen. Then, BLAST resources in NCBI [16] were used to assess the degree of specificity of the sequence recognition for these siRNAs. We selected the siRNA that reported the target gene as the only mRNA hit. To further assess siRNA specificity for S100A4, we determined the mRNA levels of Enolase 2, Topoisomerase II, Clusterin and UGT1A7 upon incubation with siS100A4 (off-target effects) by Real-Time PCR using SYBR Green dye. See Additional file 1: Primers for off-target effects determination.

### Transfection of an expression vector encoding for β-catenin

Cells were seeded into 6-well plates. Eighteen hours later, transfection with the expression plasmid for β-catenin (pcDNA3-β-catenin) kindly provided by Dr. Duñach, Universitat Autònoma de Barcelona, Spain) was performed as described for pCMV-S100A4. Cells were collected 48 h after transfection for RNA determination. S100A4 mRNA levels were monitored by RT-Real-Time PCR. The empty vector was transfected in parallel with pcDNA3-β-catenin and was used as negative control.

### Statistical analyses

Data are presented as mean ± SE. Statistical analyses were performed using the unpaired t test option in GraphPad InStat version 3.1a for Macintosh. p-values of less than 0.05 were considered statistically significant.

## Results

### S100A4 is overexpressed but not amplified in human cells resistant to methotrexate

In previous studies, we used the HG U133 PLUS 2.0 microarrays from Affymetrix as a tool to analyze the differential gene expression between sensitive and MTX-resistant cells derived from different human cell lines representative of colon cancer (HT29 and CaCo-2), breast cancer (MCF-7 and MDA-MB-468), pancreatic cancer (MIA PaCa-2), erythroblastic leukemia (K562) and osteosarcoma (Saos-2) (GEO series accession number [GSE16648], which also contains the current data). Now we performed a global analysis of the seven cell lines to find out genes differentially expressed in at least four out of the seven cell lines. The list of these genes is provided in Table 1. Among them, S100A4 was a gene differentially expressed in five out of the seven cell lines and previously reported to have a role in chemotherapy resistance [13]. We validated S100A4 overexpression in HT29, MCF-7, MIA PaCa-2, K562 and Saos-2 resistant cell lines at the levels of mRNA and protein, and determined that S100A4 upregulation in the resistant cells was not due to changes in its gene copy number (Table 2).

**Table 1:** Genes differentially expressed in at least four out of the seven MTX-resistant cell lines studied.

Genbank	Gene Name	Description	Fold Change						
			
			HT29	Caco-2	MCF-7	MDA- **MB-468**	MIA **PaCa-2**	K562	SaOs-2
U05598	AKR1C2	aldo-keto reductase family 1, member C2	4.6	10.2	0.03	31.1	0.02	5.0	NS

AB018580	AKR1C3	aldo-keto reductase family 1, member C3	1.8	4.6	0.8	73.3	0.7	12.0	NS

NM_000691	ALDH3A1	aldehyde dehydrogenase 3 family, member A1	2.9	3.8	6.6	11.9	0.4	NS	NS

AL136912	ATG10	ATG10 autophagy related 10 homolog	8.7	NS	NS	1.5	2.1	10.1	2.0

U36190	CRIP2	cysteine-rich protein 2	2.7	1.3	1.9	NS	2.2	NS	0.3

NM_001321	CSRP2	cysteine and glycine-rich protein 2	2.0	0.3	2.7	NS	2.0	2.9	0.5

AU144855	CYP1B1	cytochrome P450, family 1, subfamily B, polypeptide 1	3.1	2.3	0.5	NS	4.2	NS	0.4

AI144299	DHFR	dihydrofolate reductase	7.3	50.2	52.8	1.8	16.9	17.8	8.9

AA578546	DHFRL1	dihydrofolate reductase-like 1	7.0	10.8	5.5	NS	3.0	17.5	2.1

NM_012242	DKK1	dickkopf homolog 1 (Xenopus laevis)	4.3	2.5	2.1	NS	0.7	NS	19.1

AI745624	ELL2	elongation factor, RNA polymerase II, 2	NS	1.7	0.4	2.6	8.0	2.3	2.7

AF052094	EPAS1	endothelial PAS domain protein 1	2.8	NS	2.4	NS	6.4	0.4	0.1

AU144565	EPB41L4A	erythrocyte membrane protein band 4.1 like 4A	1.6	1.3	1.7	1.4	2.3	4.4	4.7

AW246673	FAM46A	family with sequence similarity 46, member A	NS	2.6	NS	NS	3.9	2.5	1.5

NM_000142	FGFR3	fibroblast growth factor receptor 3	NS	4.6	3.1	0.4	1.5	0.4	3.4

NM_018071	FLJ10357	hypothetical protein FLJ10357	NS	3.8	NS	NS	2.1	3.2	3.5

BE550452	HOMER1	homer homolog 1 (Drosophila)	NS	NS	27.9	NS	2.4	13.8	NS

AF243527	KLK5	kallikrein-related peptidase 5	2.3	NS	2.2	NS	3.5	NS	4.6

AK022625	LOC92270	V-type proton ATPase subunit S1-like protein	54.0	2.3	NS	NS	3.4	25.0	NS

BC006471	MLLT11	myeloid/lymphoid or mixed-lineage leukemia	2.2	2.6	NS	NS	1.8	0.08	1.7

NM_002439	MSH3	mutS homolog 3 (E. coli)	6.3	8.2	3,8	NS	13.2	35.9	1,5

NM_006117	PECI	peroxisomal D3, D2-enoyl-CoA isomerase	7.5	2.6	1.9	NS	NS	NS	1.8

BF432873	PSMD11	proteasome 26 S subunit, non-ATPase, 11	NS	2.3	NS	NS	1.3	3.5	1.4

NM_002923	RGS2	regulator of G-protein signaling 2, 24 kDa	1.6	8.2	3.6	NS	3.2	1.9	NS

AW043594	RPS23	ribosomal protein S23	12.9	NS	NS	NS	2.9	8.9	1.8

NM_005978	S100A2	S100 calcium binding protein A2	0.5	0.3	7.1	NS	4.0	NS	4.1

NM_002961	S100A4	S100 calcium binding protein A4	3.7	0.6	4.3	NS	2.4	1.5	2.5

AL565362	SLC2A13	solute carrier family 2, member 13	4.0	9.4	0.6	2.5	4.3	10.2	NS

BG150485	SLC6A6	solute carrier family 6, member 6	0.6	2.6	2.2	NS	2.4	1.5	1.5

NM_003069	SMARCA1	SWI/SNF related, matrix associated, actin dependent regulator of chromatin, subfamily A, member 1	2.6	2.6	14.0	NS	NS	3.4	1.7

NM_012446	SSBP2	single-stranded DNA binding protein 2	10.6	NS	0.5	NS	2.9	5.0	2.0

AW134979	STXBP6	syntaxin binding protein 6 (amisyn)	NS	2.1	27.5	NS	3.0	14.5	0.5

NM_003248	THBS4	thrombospondin 4	NS	12.9	9.2	NS	15.7	4.9	0.6

BE873420	UGT1A6	UDP glucuronosyltransferase 1, polypeptide A6	NS	2.8	24.4	27.9	5.3	NS	NS

AI922599	VIM	vimentin	2.4	NS	3.6	NS	NS	4.9	1.7

BE539792	ZCCHC9	zinc finger, CCHC domain containing 9	8.3	1.5	0.5	NS	6.7	7.9	NS

BU078629	ZFYVE16	zinc finger, FYVE domain containing 16	6.1	45.8	76.4	NS	16.5	23.1	3.2

A global comparison analysis was performed to find out differentially expressed genes in the majority of the MTX-resistant cells. The table includes the list of the genes differentially expressed in at least four out of the seven MTX-resistant cell lines that displayed a Benjamini-Hochberg false discovery rate-corrected p-value < 0.05. For each gene, the Genbank accession number, gene name, description and fold change in each resistant cell line with respect to its respective sensitive counterpart are shown. All values displayed are significant, unless otherwise stated (NS, non significant; p-value > 0.05)

**Table 2:** Validation of S100A4 overexpression and copy number determination in the different cell lines.

Cell Line	Expression					
			
	Microarray					
				
	Signal Sensitive	Signal Resistant	Ratio	RT-PCR Validation	Copy- number	Protein
HT29	1876	6862	3.7	6.2 ± 0.2	1.4 ± 0.1	22.5 ± 1.0

MCF-7	49	213	4.3	6.2 ± 0.3	1.5 ± 0.1	10.8 ± 2.2

MiaPaCa-2	3304	7925	2.4	4.7 ± 0.6	1.2 ± 0.1	24.4 ± 8.1

K562	147	249	1.5	3.6 ± 0.2	1.6 ± 0.1	4.0 ± 1.0

Saos-2	137	345	2.5	2.2 ± 0.1	2.16 ± 0.07	2 ± 0.4

CaCo-2	4515	2769	0.6	N/D	N/D	N/D

MDA-MB-468	317	235	0.7	N/D	N/D	N/D

The table shows the S100A4 expression levels obtained in the microarray experiments for all the cell lines studied. The level of expression is presented as the mean of the signal value in sensitive and resistant cells, as well as the ratio between them (fold change). Gene overexpression was validated in five of the seven cell lines by RT-Real-Time PCR. *S100A4 *gene copy number was determined by Real-Time PCR. Protein levels were determined by Western blot using a specific antibody, and quantitation was performed upon densitometry of the images. Values represent the mean (in fold change relative to each respective sensitive cells) of three experiments ± SE; N/D, non-determined.

### Ectopic overexpression of S100A4 desensitizes HT29 cells toward MTX

**Figure 1:**
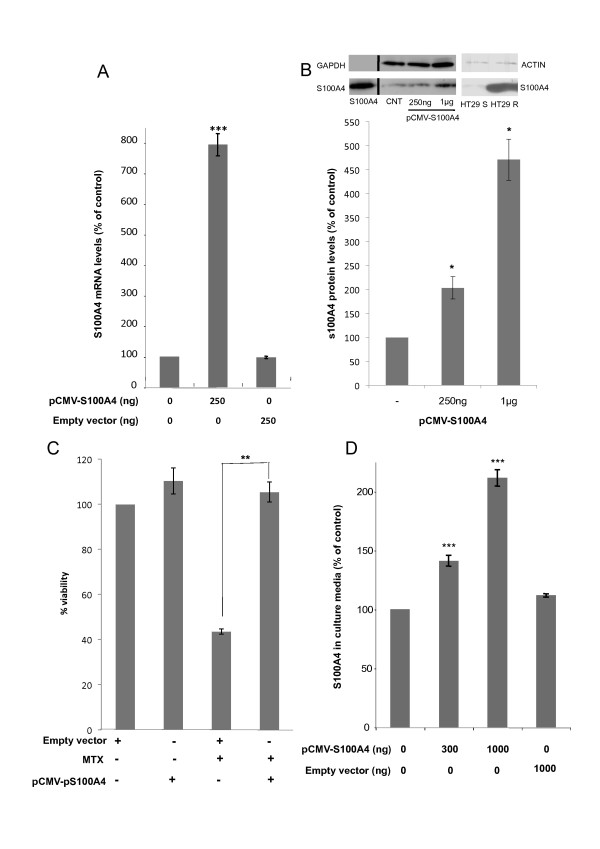
**Effects on S100A4 expression and MTX sensitivity upon pCMV-S100A4 transfection of HT29 cells**. A) mRNA levels of S100A4 determined by RT-Real-Time PCR 48 h after treatment of HT29 cells (30,000) with 250 ng of the expression vector for S100A4 (pCMV-S100A4). B) A representative image of the intracellular protein levels of S100A4 determined by Western Blotting 72 h after ectopic transfection with its expression vector is shown in the upper panel, and the quantification of the blots is shown in the lower panel. Purified S100A4 protein was used as a reference marker (Abnova; first lane). An additional panel showing endogenous S100A4 protein levels in HT29 sensitive (S) and resistant (R) cells is also provided. C) Effects of S100A4 overexpression on cell viability. HT29 cells (100,000) were treated with 1 μg of pCMV-S100A4 and 5 × 10^-8^M MTX was added 48 h later. Cell viability was assessed by the MTT assay six days after MTX treatment. D) Extracellular S100A4 protein levels quantified by ELISA 72 h after S100A4 overexpression upon pCMV-S100A4 transfection. The expression and viability results are expressed as percentages referred to the untreated cells. Values are the mean of three independent experiments ± SE. *p < 0.05, **p < 0.01, *** p < 0.001.

HT29 MTX-resistant cells displayed the highest S100A4 expression values, considering both the mRNA and protein levels (Table 2). Thus, HT29 cells were selected for further studies using an expression vector for S100A4 (pCMV-S100A4). Transfection with 250 ng of this vector produced a 8-fold increase in the levels of S100A4 RNA after 48 hours (Figure 1A). The intracellular levels of S100A4 protein were also increased, reaching almost 5 fold when transfecting 1 μg of the expression plasmid (Figure 1B). The overexpression of S100A4 was associated to a decrease in the sensitivity toward MTX (Figure 1C).

### S100A4 is secreted by HT29 cells transfected with pCMV-S100A4

Given that it had been described that S100A4 has extracellular functions [12,17], we explored S100A4 protein levels in the culture media of pCMV-S100A4 transfected cells. The results from the ELISA assays showed increased amounts of S100A4 protein in media from transfected cells (Figure 1D), thus indicating that a fraction of the protein produced upon vector transfection was secreted outside the cells.

### Knocking down S100A4 with siRNA chemosensitizes HT29 toward MTX

**Figure 2:**
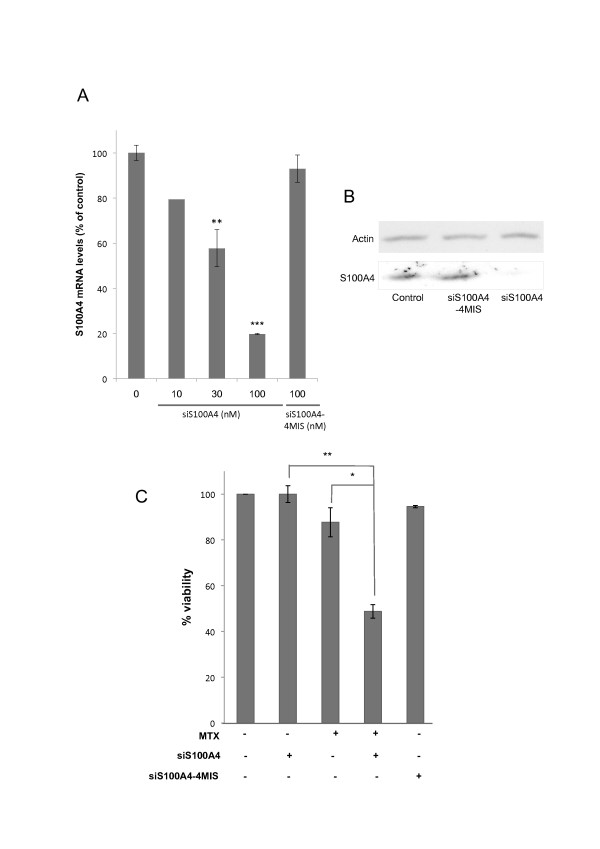
**Effects on S100A4 expression and MTX sensitivity upon siS100A4 transfection of HT29 cells**. A) HT29 cells (30,000) were transfected with siS100A4 as described in Methods. Total RNA was extracted after 48 h and S100A4 mRNA levels were determined by RT-Real-Time PCR. B) S100A4 protein levels were determined by Western Blotting 72 h after transfection, using specific antibodies against S100A4 and Actin to normalize the results. C) Chemosensitization assays toward methotrexate: cells were treated with siS100A4 for 48 h and then incubated with MTX. Cell viability was determined 3 days after MTX treatment. The expression and viability results are expressed as percentages referred to the untreated cells. Values are the mean of three independent experiments ± SE. A representative image of Western Blots is presented. *p < 0.05, ** p < 0.01, *** p < 0.001.

We used iRNA technology to study the role of S100A4 in MTX resistance. Treatment of sensitive HT29 cells with increasing concentrations (10-100 nM) of the siRNA against S100A4 (siS100A4) showed a progressive decrease in its mRNA levels (Figure 2A), causing an 80% reduction at 100 nM. This treatment also caused a vast decrease in S100A4 protein levels (Figure 2B) and increased the sensitivity of HT29 cells toward MTX by about 50% (Figure 2C). To further assess siRNA specificity for S100A4, we determined the mRNA levels of other cellular genes upon incubation with siS100A4 (off-target effects). These experiments did not show any significant variation of the mRNA levels for Enolase 2, Topoisomerase II, Clusterin and UGT1A7 (Table 3)

**Table 3:** Assessment of siS100A4 specificity.

Gene	S100A4	Enolase 2	Topoisomerase II	Clusterin	UGT1A7
Control	100	100	100	100	100
siRNA S100A4	18.6 ± 6	135.8 ± 21.6	127.5 ± 6.4	133.9 ± 14.4	159.5 ± 28.4

HT29 cells were transfected with 100 nM siS100A4. Forty-eight hours later, the mRNA levels of S100A4 and four unrelated genes were determined by RT-Real-Time PCR. Data represent the mean of three experiments ± SE.

**Figure 3:**
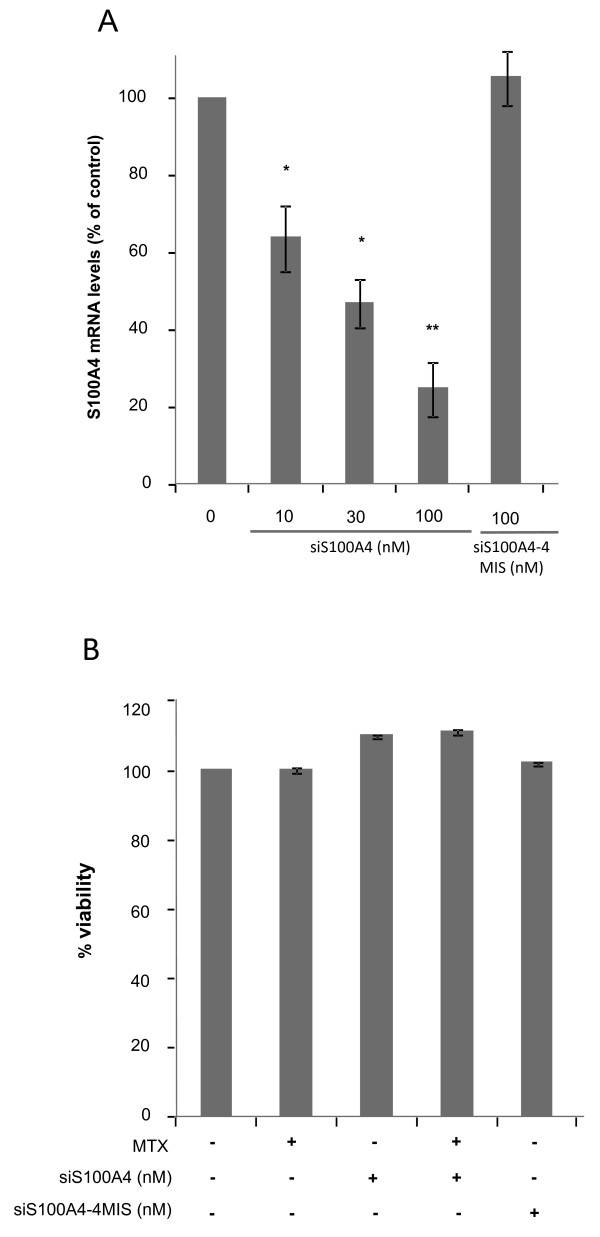
**Effects on S100A4 expression and MTX sensitivity upon siS100A4 transfection of HT29 MTX-resistant cells**. A) S100A4 mRNA levels were determined by RT-Real-time PCR as described in Methods 48 h after siS100A4 treatment. B) Cells were treated with MTX after a 48 h-pre-incubation with siS100A4, and cell viability was determined 3 days later by the MTT assay. The levels of expression and the viability results are expressed as percentages referred to the untreated cells. Values are the mean of three independent experiments ± SE. *p < 0.05, ** p < 0.01.

Treatment with 100 nM siS100A4 was also performed in HT29 MTX-resistant cells. This approach led to a reduction of 75% in the levels of S100A4 RNA (Figure 3A) but had no effect on cell viability (Figure 3B). A 4-mismatch siRNA against S100A4 (siS100A4-4 MIS) was used as negative control, and did not cause any significant effects on S100A4 mRNA, protein levels or cell viability, in neither of the two cell lines.

### S100A4 is transcriptionally regulated by the Wnt pathway in HT29 cells

**Figure 4:**
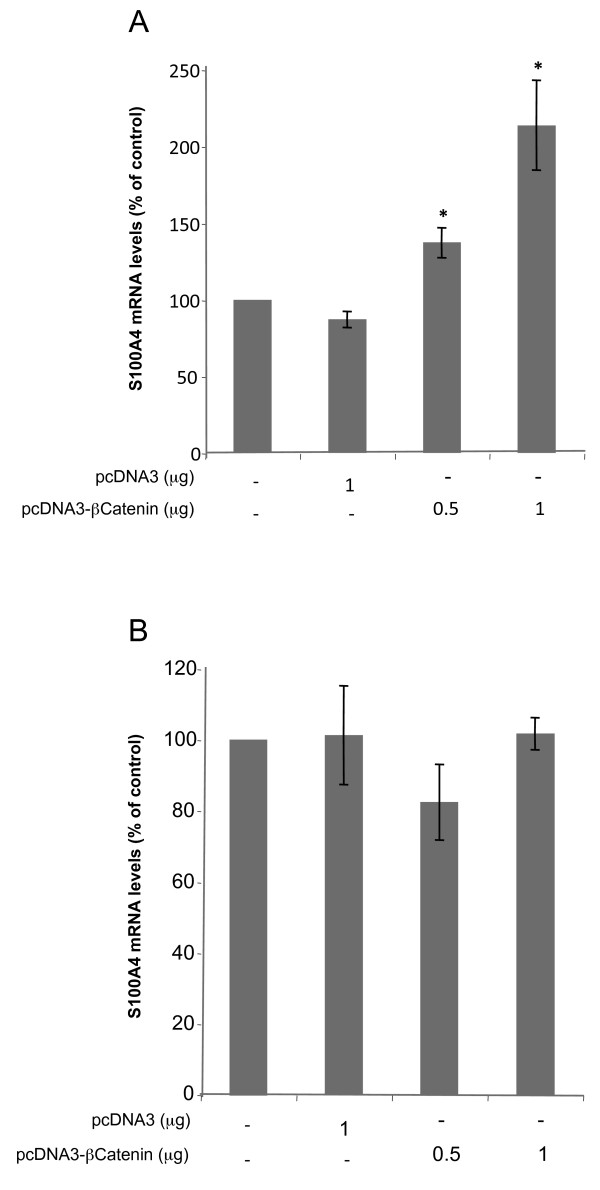
**Effects of transfecting an expression vector encoding for β-Catenin on S100A4 mRNA levels**. Transfection with β-Catenin expression vector (pcDNA3-β-Catenin) was performed in HT29 cells, both sensitive (Figure 4A) and resistant (Figure 4B) as described in Methods. S100A4 mRNA levels were determined by RT-Real-Time PCR 48 h after transfection. All results are expressed as percentages referred to untreated cells. Values are the mean of three independent experiments ± SE. * p < 0.05.

It had been previously described that S100A4 was a target of the Wnt signaling pathway and a functional TCF binding site has been identified in its promoter sequence [18]. To investigate whether S100A4 expression was regulated through this pathway in HT29 cells, we overexpressed β-catenin in HT29 sensitive or resistant cells and quantified S100A4 mRNA levels 48 h after transfection. A 2-fold increase in S100A4 mRNA levels was observed upon transfection of 1 μg of pcDNA3-β-catenin in HT29 sensitive cells (Figure 4A) whereas no changes were obtained when transfection was performed in HT29 resistant cells (Figure 4B).

## Discussion

The principal aim of this work was to find out genes differentially expressed in cell lines resistant to MTX representative of five different types of human cancer. In a previous report [3], we determined and compared the patterns of differential gene expression associated to MTX resistance in seven human cancer cell lines. In that work, we established that the only differentially expressed gene in common among all the cell lines studied was DHFR. In the present work, we identified genes differentially expressed in at least four out of the seven cell lines studied. Among the genes that fulfilled this requisite, we found some genes that we had previously validated and reported to be directly associated with MTX resistance in HT29 MTX-resistant cells (DHFR, AKR1C1 and DKK1) or suggested to contribute to the phenotype in this cell line (MSH3, SSBP2 and ZFYVE16) [3-5]. We also found UGT1A6, a gene that seems to play a role in MTX resistance in MCF-7 and in MDA-MB-468 cells (data not shown). Among the other genes in the list, we selected, for expression, genomic and functional validation, S100A4, a gene overexpressed in five out of these seven cell lines that was the only one previously reported to be associated with chemoresistance [13]. This gene has also been related with malignancy, metastasis, and tumor progression.

The human S100A4 is a member of the S100 family of EF-hand Ca^2+^-binding proteins. The multigenic family of S100 proteins is expressed in vertebrates exclusively and has been described to play intracellular and extracellular regulatory activities on protein phosphorylation, on the dynamics of cytoskeleton components or on Ca^2+ ^homeostasis [8,9]. S100A4 in particular has been described to have a function in cell cycle progression, in cell motility and as a modulator of intercellular adhesion and of the invasive properties of cells [9,19] (see Sherbet et al. for a review [20]). Several cancers, including colon cancer, breast cancer and osteosarcoma, are known to produce S100A4 [10], and particularly, HT29 cells have been described to express a substantial amount of S100A4 [21]. Overexpression of S100A4 is more frequently found in cancer cells than in normal colonic mucosa, as well as more in liver metastasis than in primary tumors [21]. A correlation between S100A4 expression levels and the invasive potential of HT29 cells has been suggested [21]. S100A4 gene overexpression has been related to the malignant potential of some tumors [10] and has been closely associated with metastasis in several human cancers, including colorectal [21], breast [22,23], ovary [24], thyroid [25], pancreatic [26], lung [27,28], esophageal [29], prostate [30] and gastric cancer [31,32].

We tried to mimic the overexpression of S100A4 in the resistant cells by transfecting HT29 sensitive cells with an expression vector encoding for S100A4. We tested that S100A4 mRNA and intracellular protein levels were significantly increased after transfection. Importantly, we could observe a reversion of the cytotoxicity caused by MTX upon transfection with S100A4 expression vector. It is worth mentioning that a moderate S100A4 overexpression has been described in a colon cancer cell line resistant to doxorubicin [33]. S100P, another member of the S100 family, has also been related to doxorubicin resistance in colon cancer [33]. Moreover, S100A4 expression has been detected in patients that had received adjuvant 5-fluorouracil, and has been proposed to be a predictive factor of relapse in gastric cancer [34]. Likewise, S100P overexpression has been reported to promote pancreatic cancer growth and increase cell survival after 5-fluorouracil exposure [35].

To investigate the role of intracellular S100A4 on MTX resistance, we used iRNA technology. Transfection of a siRNA against S100A4 in HT29 MTX-resistant cells caused a reduction in gene RNA levels but did not alter cell viability. The latter effect could be explained by the overexpression by gene amplification of the *dhfr *locus in this resistant cell line, which would mask the effects of the siRNA used. However, when the same treatments were performed in HT29 sensitive cells, carrying no *dhfr *amplification, we could achieve a chemosensitization of these cells toward MTX. These latter results are in agreement with Mahon *et al*. [13] that showed that inhibition of S100A4 expression resulted in an increased sensitivity of pancreatic ductal adenocarcinoma cell lines to gemcitabine treatment and induced apoptosis. It has also been described that S100A4 knockdown enhances the sensitivity of osteocarcinoma cells to undergo apoptosis [36] and reverses the metastatic potential of osteosarcoma [37] and lung carcinoma cells [28]. These reports together with our results using either the expression vector or siRNA technology, provide evidence that S100A4 acts as a pro-survival factor that contributes, together with DHFR, AKR1C1 and DKK1 (see above), to chemoresistance in HT29 MTX-resistant cells. S100A4 could be considered as biomarker of resistance in the sense that it is overexpressed in the majority of the resistant cell lines analyzed, and its determination is easy to perform. In addition it could be considered as a drug target in the category of resistance modulator using inhibitors of S100A4 as an adjuvant in MTX treatment.

In an attempt to determine the mechanism for the overexpression of S100A4 in HT29 resistant cells, we determined S100A4 copy number in both HT29 cell lines. Although it has been previously described that the chromosomal region where the gene is located (1q21) is amplified in breast cancer [38,39] and in tumor tissue from osteosarcoma patients [40], we did not observe copy number changes for S100A4 in any of the cell lines studied. Other mechanisms have been proposed that attempt to explain the mechanism for S100A4 overexpression: i) by hypomethylation, since increased S100A4 expression levels have been associated with decreased methylation of the first intron of this gene in cell lines and carcinomas of pancreatic origin [26] and of the second intron in colon cancer cell lines [41]; and ii) through the Wnt signaling pathway, given that it has been shown that S100A4 is a target of this pathway in colon cancer and a functional TCF binding site has been identified in its promoter sequence [18]. In this sense, we had previously proposed the activation of the Wnt/β-catenin pathway to be an important step in MTX resistance in HT29 colon cancer cells [5]. In this cell line, E-cadherin is lost at the chromosome level and underexpressed, thus allowing β-catenin to play its function in gene transcription. In this direction, an inverse correlation has been established between the expression levels of S100A4 and E-Cadherin [42] and has been associated with poor differentiation of cancer cells [31]. Moreover, transfection of an E-Cadherin expression vector has been reported to cause a decrease in S100A4 expression levels [43]. To further elucidate if the Wnt signaling pathway could be responsible for the increased expression levels of S100A4 in the resistant cells, we overexpressed β-catenin and determined S100A4 mRNA levels. The resistant cells, which already have the Wnt/β-catenin pathway highly activated, do not respond to β-catenin overexpression. However, the results obtained in HT29 sensitive cells showing a two-fold increase in S100A4 mRNA expression upon β-catenin transfection give support to a Wnt/β-catenin pathway-mediated S100A4 transcription. Another target of the Wnt signaling pathway that had been previously related with MTX resistance is P-glycoprotein [44,45]. However, no changes in its expression levels were evidenced by our microarray results in HT29 cells (data not shown).

Although many biological functions have been attributed to S100A4, the exact molecular mechanisms by which S100A4 exerts these functions have not been fully elucidated [46]. Extracellular S100A4 has been related to metastasis and angiogenesis [17,47,48] and has been reported to activate NF-κB signaling. S100A4-mediated activation of NF-κB has been proposed to repress the expression of BNIP3, a pro-apoptotic member of the Bcl-2 family, leading to decreased apoptosis [13] and increased resistance to chemotherapy [49]. Our ELISA experiments demonstrated that S100A4 ectopic overexpression in the sensitive cells leads to the secretion of a fraction of the synthesized protein. Secretion of S100A4 by tumor cells has been previously demonstrated *in vitro *[12]. One can hypothesize that secreted S100A4 would contribute to resistance through BNIP3 regulation by the NF-κB pathway in HT29 cells. However, our microarray results show no changes in BNIP3 expression levels in HT29 resistant cells (data not shown), thus suggesting that this gene does not play a role in chemoresistance in this cell line.

S100A4 has been reported to interact with p53, thus interfering in p53 activation and DNA binding capacity [50]. Mutations in p53 are frequently found in colorectal cancer [51], and HT29 cells bear mutations in this gene. These mutations reduce p53-dependent responses, thus allowing the cells to continue to proliferate and therefore give rise to drug resistance [52,53]. To further establish MTX resistance, interaction between p53 and S100A4 would be necessary to reduce p53 remaining activity. The significant 2-fold decrease in p53 expression levels in HT29 resistant cells shown in our microarray results, in accordance with others [54], would lead to increased proliferation and reduced apoptosis, both of which would contribute to MTX resistance.

In summary, our results show a role for S100A4 in MTX resistance. Its overexpression in HT29 MTX-resistant cells is not due to changes in gene copy-number but to a transcriptional regulation, probably through the Wnt pathway. Cellular knockdown of S100A4 leads to chemosensitization toward MTX and ectopic gene overexpression desensitizes the cells toward this chemotherapeutic agent.

## Conclusions

S100A4 was identified and confirmed as a gene overexpressed in five out of the seven MTX-resistant cell lines studied. S100A4 ectopic overexpression in HT29 cells lead to a desensitization toward MTX. Conversely, transfection experiments using interference RNA produced a chemosensitization to MTX. The results obtained in this report establish a relation between S100A4 and resistance to MTX.

## Abbreviations

MTX: methotrexate; DHFR: dihydrofolate reductase; S100A4: S100 calcium binding protein A4.

## Competing interests

The authors declare that they have no competing interests.

## Authors' contributions

NM, ES, CA, XV, IR, JA, SR, and JLH performed the experimental work. ES and CJC analyzed the data. VN and CJC designed the study, helped with data interpretation and supervised the experimental work. All authors wrote and approved the final manuscript.

## Pre-publication history

The pre-publication history for this paper can be accessed here:

http://www.biomedcentral.com/1471-2407/10/250/prepub

## Supplementary Material

Additional file 1 **Primers for off-target effects determination**. The word table shows the sequences of the primers used to assess the off-target effects of transfected siS100A4 by determining the mRNA levels of Enolase 2, Topoisomerase II, Clusterin and UGT1A7 by RT-Real-Time PCR. APRT mRNA was used to normalize the results. Click here for file
