# Supplementary material for: Overexpression of S100A4 in human cancer cell lines resistant to methotrexate
Source: BMC Cancer. 2010 Jun 1;10:250. doi: 10.1186/1471-2407-10-250 (PMC2903526; doi:10.1186/1471-2407-10-250)
Supplement: Additional file 1 — Primers for off-target effects determination. The word table shows the sequences of the primers used to assess the off-target effects of transfected siS100A4 by determining the mRNA levels of Enolase 2, Topoisomerase II, Clusterin and UGT1A7 by RT-Real-Time PCR. APRT mRNA was used to normalize the results. [file 1471-2407-10-250-S1.DOC]

| **Gene** | **Primers sequence** |
| --- | --- |
| Enolase 2 | For 5'- TGCCTCAGAGTTTTATCGTG -3'  Rev 5'- CTTGAGCAGCAGACAGTTG -3' |
| Topoisomerase II | For 5’-GAGAAGGACCGGGAAAAGTC-3’  Rev 5’-GCCATCATCTTCAGGTTCATC-3’ |
| UGT1A7 | For 5’-CTTTGCCAAGGCAGGGAAG  Rev 5’-AGAAAATGCACTTCGCAATGG-3’ |
| CLUSTERIN | For 5’-CGGCGGGAGCTCGACGAATC-3’  Rev 5’-CCTGCAGCGCTTTCTCCGC-3’ |
| APRT | For 5’-GCAGCTGGTTGAGCAGCGGAT-3’  Rev 5’-AGAGTGGGGCCTGGCAGCTTC-3’ |
